# Antimicrobial Susceptibility Patterns and Resistance Trends of *Staphylococcus aureus* and Coagulase-Negative Staphylococci Strains Isolated from Ocular Infections

**DOI:** 10.3390/antibiotics10050527

**Published:** 2021-05-03

**Authors:** Francesco Petrillo, Danilo Pignataro, Federica Maria Di Lella, Michele Reibaldi, Matteo Fallico, Niccolò Castellino, Guglielmo Parisi, Maria Consiglia Trotta, Michele D’Amico, Biagio Santella, Veronica Folliero, Maria Teresa Della Rocca, Michele Rinaldi, Gianluigi Franci, Teresio Avitabile, Marilena Galdiero, Giovanni Boccia

**Affiliations:** 1 Section of Ophthalmology, University Hospital “Policlinico-Vittorio Emanuele”, 95123 Catania, Italy; francescopetrillo09@gmail.com (F.P.); matteofallico@hotmail.com (M.F.); ncastellino7@gmail.com (N.C.); t.avitabile@unict.it (T.A.); 2 Section of Microbiology and Virology, University Hospital “Luigi Vanvitelli”, 80138 Naples, Italy; Danilopignataro.89@gmail.com (D.P.); federicamariadilella@gmail.com (F.M.D.L.); bi.santella@gmail.com (B.S.); veronica.folliero@unicampania.it (V.F.); mariateresa.dellarocca@unicampania.it (M.T.D.R.); 3 Department of Surgical Sciences, Eye Clinic Section, University of Turin, 10122 Turin, Italy; mreibaldi@libero.it (M.R.); guglielmoparisi@gmail.com (G.P.); 4 Department of Experimental Medicine, Division of Pharmacology, University of Campania “Luigi Vanvitelli”, 80138 Naples, Italy; mariaconsiglia.trotta2@unicampania.it (M.C.T.); michele.damico@unicampania.it (M.D.); 5 Department of Ophthalmology, University Hospital “Luigi Vanvitelli”, 80138 Naples, Italy; michele.rinaldi@unicampania.it; 6 Department of Medicine, Surgery and Dentistry “Scuola Medica Salernitana”, University of Salerno, 84081 Baronissi, Italy; gfranci@unisa.it

**Keywords:** eye infections, bacterial, methicillin-resistant staphylococcus aureus, drug resistance, hospitals

## Abstract

Ocular bacterial infections represent a serious problem that affecting people of all age and genders. These infections can lead to visual impairment and blindness if not properly treated. The current study evaluates the antimicrobial resistance profiles and the resistance trend of both *Staphylococcus aureus* (*S. aureus*) and coagulase-negative staphylococci (CoNS), the main pathogens involved in eye infections. A total of 322 isolates of *S. aureus* and CoNS, were collected from patients with bacterial conjunctivitis and keratitis at the “Luigi Vanvitelli” University Hospital of Campania in Naples, Italy, between 2017 and 2020. The isolated bacteria showed a high percentage of resistance to methicillin and other antibiotics commonly used for the treatment of ocular infections. Trends in antibiotic resistance were not encouraging, recording—especially among CoNS strains—an increase of more than 20% in resistance to methicillin and aminoglycosides during the study period. Instead, the resistance rates to tetracycline had a significant decrease in CoNS isolates while no changes in their susceptibility to fluoroquinolones and macrolides were observed. However, all isolates showed no resistance to trimethoprim/sulfamethoxazole and chloramphenicol. In this scenario, preventive identification of the infection causative agents and the evaluation of the antimicrobial susceptibility patterns are essential to set up an ocular infection effective drug treatment and also prevent antibiotic resistance.

## 1. Introduction

Although the ocular surface is invariably exposed to a wide range of microorganisms, the eye is generally impermeable to almost all potentially infectious external agents, relying on a large number of natural defense mechanisms, such as the presence of a commensal microbial flora—capable of preventing the engraftment of pathogenic microorganisms—and the tear film—containing agents such as lactoferrin, defensins and lysozyme, with high antimicrobial power [1]. However, some conditions, such as prolonged and improper use of contact lenses, surgery, trauma, previous ocular infections, dry eye state, nasolacrimal duct obstructions or reduced host defense, can cause several ocular infections by adapted microorganisms [2,3]. Bacteria are responsible for about 32–74% of ocular infections, followed by viruses, fungi and parasites [4,5]. Worldwide people of all age and genders can have bacterial eye infections that include keratitis, dacryocystitis, endophthalmitis, blepharitis and conjunctivitis [6,7]. These ophthalmic infections, if not properly treated, can alter the normal structure of the eye, leading to visual impairment and blindness. As reported in several studies, the most common bacteria involved in ocular infections are *Staphylococcus aureus* (*S. aureus*), Coagulase-negative staphylococci (CoNS), *Streptococcus pneumoniae*, *Corynebacterium spp.*, *Bacillus spp., Nocardia, Pseudomonas aeruginosa, Haemophilus influenzae* and *Enterobacteriaceae* [8]. Among these bacteria, *S. aureus* and CoNS strains are very important for their high prevalence of infection, while *S. aureus* is among the most common cause of blepharitis, conjunctivitis, dacryocystitis, keratitis and endophthalmitis. CoNS strains, although considered to be part of the commensal skin flora, represent a source of infection when patients show clinical symptoms, inflammation and risk factors such as previous surgical intervention, implanted foreign bodies and local/systemic immunosuppression. Several studies have found the high prevalence of *S. aureus* and CoNS strains in conjunctivitis, keratitis, endophthalmitis and blepharitis. In Ethiopia, the prevalence of *S. aureus* and CoNS strains was 50.3% and 33.5%, respectively [9]. A similar study conducted in Florida reported that *S. aureus* and CoNS strains were isolated in 4.7% and 62.8% of cases [10]. The high prevalence of CoNS strains in ocular samples is particularly associated with invasive interventions and the use of permanent or implanted ocular devices, essential in modern clinical practice [9]. The most used antibiotic classes for eye infection treatment are penicillins, aminoglycosides, fluoroquinolones, tetracyclines, macrolides, phenicols and sulfonamides [11,12]. However, these treatments have become complicated due to the rise of bacterial strains resistant to different antimicrobial agents. This is particularly true for *S. aureus* and CoNS eye infections, in which the treatment is often difficult due to methicillin resistance (MR). Several studies reported antimicrobial resistance (AMR) among *Staphylococcus species*. Among these, The National Surveillance Study (ARMOR) has monitored eye infections since 2009 for bacterial species resistance profiles [13,14]. In particular, MR-*Staphylococcus* strains showed not only β-lactam antibiotic resistance but also a resistance profile to other antimicrobial agents commonly used in the treatment of ocular infections The improper, excessive or empirical use of antimicrobial agents increased the resistance profile pattern. In this scenario, the increasing antimicrobial resistance by microorganisms involved in ocular infections required surveillance in order to guide empirical therapy [15]. The aim of the present study was to evaluate the antibiotic susceptibility patterns, resistance rates and trend of these Gram-positive bacteria isolated from patients with bacterial conjunctivitis and keratitis at University Hospital of Campania “Luigi Vanvitelli” in Naples, Italy.

## 2. Results

During the study period, 322 non-repetitive *Staphylococcus* strains were isolated from patients with bacterial conjunctivitis and keratitis clinically diagnosed and laboratory confirmed at the “Luigi Vanvitelli” University Hospital of Campania in Naples, Italy. Among these isolated strains, 49.1% were *S. aureus*, while 50.9% were CoNS (*Staphylococcus epidermidis*, *Staphylococcus hominis*, *Staphylococcus xylosus*, *Staphylococcus haemolyticus* and *Staphylococcus warneri*). These percentages suggested that eye infections were sustained by *S. aureus* and CoNS in equal measure. No statistically significant differences were observed between male (48.1%) and female (51.9%). Instead, the rate of staphylococci ocular infections was higher in the group of patients over 60 years old (66.8%), while the least infection cases were observed in the group under 31 years of age (8.0%). There were no significant differences between eye infections caused by *S. aureus* and CoNS with respect to gender or different age groups. Gender and age groups distribution are shown in Table 1.

In this study, the antimicrobial susceptibility patterns of *S. aureus* and CoNS ocular isolates were analyzed. Most of these Gram-positive isolates had shown a high MR rate. In particular, among the *S. aureus* strains, 23.7% had an MR-phenotype (MRSA), while, among the CoNS isolates, 61.7% had shown MR-resistance. The rate of MR-CoNS-induced ocular infections was significantly higher than that of *S. aureus* (*p* < 0.05). Moreover, among all MR-isolates, 297 (92.5%) demonstrated a greater and significant macrolide-lincosamide-streptogramin B (MLSB) resistance phenotype. Of these, 67% were MR-CoNS and only 33% were represented by MRSA strains. Most MR-CoNS isolates (36.2%) had shown an inducible MLSB (MLSBi) resistance. All *Staphylococcus* isolates were susceptible to trimethoprim/sulfamethoxazole and chloramphenicol, whereas more CoNS isolates, compared to *S. aureus* strains, were significantly (*p* ≤ 0.05) resistant to quinolones, such as ciprofloxacin and moxifloxacin (84.2%), aminoglycosides, such as gentamycin, tobramycin and neomycin (72.8%), macrolides, such as erythromycin (81.9%), and also tetracycline (58.5%). Moreover, the majority of CoNS strains (60%) under study presented a simultaneous resistance to the antimicrobials mentioned above, methicillin and MLSB agents. The antibiotic resistance patterns of *S. aureus* and CoNS isolates are shown in Table 2.

**Figure 1:**
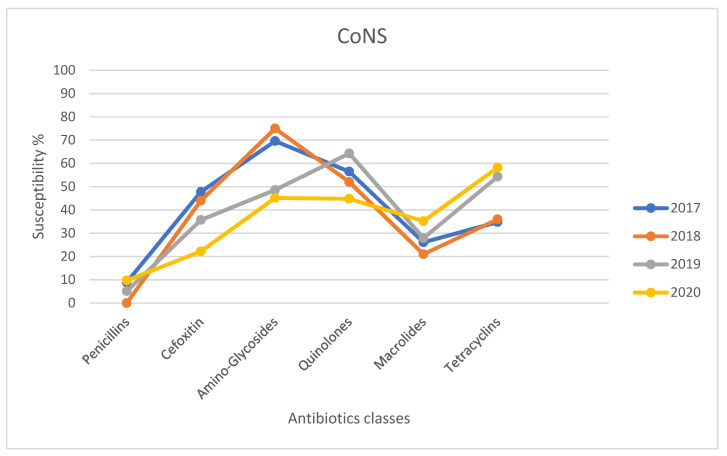
Susceptibility rates of CoNS strains for different tested antibiotics classes in 2017–2020.

**Figure 2:**
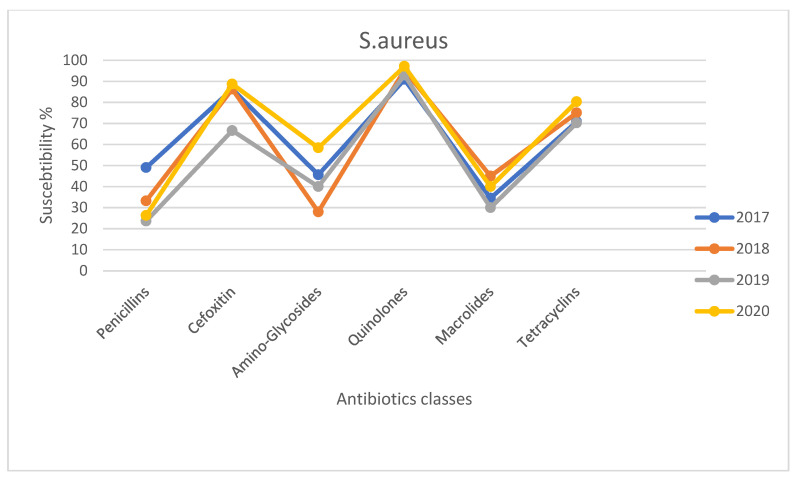
Susceptibility rates of *S. aureus* isolated strains for different tested antibiotics classes in 2017–2020.

To investigate the trend of bacterial resistance in the ocular infections, the pattern of susceptibility to several antibiotic classes by *S. aureus* and CoNS in the period of 2017–2020 was evaluated. The susceptibility rates of CoNS and *S. aureus* isolated strains for different tested antibiotic classes are shown in Figure 1 and Figure 2, respectively. The trend of resistance had remained almost unchanged in the *S. aureus* isolates between 2017 and 2020, recording only a transient but significant increase (22%) in methicillin and consequently in beta-lactamase inhibitor-resistant strains in 2019 compared to the previous two years and 2020, while the major significant variations were observed for the CoNS strains. Indeed, the susceptibility rates to methicillin and aminoglycosides in CoNS infections decreased from 47.8% to 22.2% and from 69.6% to 45.2% respectively between 2017 and 2020, thus illustrating significant reduction rates in 2020 compared to the previous 3 years (*p* < 0.05 and *p* < 0.05, respectively). In contrast, the prevalence of tetracycline resistance significantly (*p* = 0.05) decreased between 2017 and 2020. However, there was no change in the prevalence of CoNS resistant strains to fluoroquinolones and macrolides over the 4 years.

## 3. Discussion

Bacterial eye infections are frequently diagnosed, affecting people of both genders and all ages [16]. In ocular infections, the therapeutic success is associated with a timely and accurate diagnosis, together with the administration of specific antibiotics after an antibiotic susceptibility profile evaluation on the microorganisms that caused the infection [17,18,19]. To reduce antibiotic resistance, the surveillance of susceptibility patterns can guide the clinicians to appropriate empirical therapy. Given the high percentage of eye infections caused by *S. aureus* and CoNS and the increasing resistance to various antibacterial agents shown by these pathogenic microorganisms, the current analysis aims to evaluate the antibiotic susceptibility patterns, the resistance rates and the trend of these Gram-positive bacteria isolated from patients with eye infections. In this retrospective study, a total of 322 isolated strains of *S. aureus* and CoNS isolates, were collected from patients with bacterial conjunctivitis and keratitis at the “Luigi Vanvitelli” University Hospital of Campania in Naples, between January 2017 and September 2020. No significant differences were observed between male and female patients in agreement with the P. Courtright and S. K. West study [20]. On the contrary, several studies described a high incidence of ocular infections among male or female patients [21,22,23]. These gender rate variations may be due to many social factors and lifestyle habits. In our study, the most infected patients showed an age prevalence of 60–90 years old [24]. This high prevalence may be related to poor hand hygiene but also to the greater likelihood, compared to the other two age groups, of being exposed to environments such as nursing homes and hospitals, which allow rapid spread of resistant infections. A high rate of staphylococci ocular infections was also reported in India, Iran and Ethiopia [25]. Our data did not show significant differences between eye infections caused by *S. aureus* and CoNS according to K. Murugan’s study. Different results were observed in Ethiopia, Nigeria and the USA, where the most isolated strain was *S. aureus*. Instead, as described by Petrillo F. et al., a higher incidence of CoNS (60.4%) eye infections was reported among pediatric patients. CoNS were considered a part of commensal skin flora, so their presence was dismissed as sampling contamination, but in recent years, they have been reported as human pathogens that cause ocular infections in patients with clinical symptoms [4]. In the present study, methicillin-resistance was reported in 61.7% among CoNS isolates. This percentage was higher than that observed in other country, such as Ethiopia (45.2%), Uganda (27.6%) and the United States (47.4%). The most common antibiotics administrated in ocular infections are fluoroquinolones, aminoglycosides and chloramphenicol. Unluckily, our data showed that staphylococci isolates, in particular CoNS strains, were very resistant to fluoroquinolones, aminoglycosides and also tetracycline. Additionally, 60% of CoNS strains have a simultaneous resistance to the aforementioned antimicrobials, methicillin and MLSB agents, thus greatly reducing the number of available therapeutic options for treating eye infections [26]. On the contrary, all isolates were susceptible to trimethoprim/sulfamethoxazole and chloramphenicol, which could be used as choice antibiotics in difficult eye infections.

## 4. Materials and Methods

### 4.1. Sample Collection

In our retrospective study, a total of 322 isolates of *S. aureus* and CoNS, were collected from patients with bacterial conjunctivitis and keratitis at the “Luigi Vanvitelli” University Hospital of Campania in Naples, Italy, between January 2017 and September 2020. Conjunctival samples were obtained by swabbing the lower fornix of the conjunctival sac from medial to lateral and back again, using a sterile cotton swab and were processed within one hour of collection. Corneal samples were obtained by ulcer scraping at the base and at the leading edge of the infiltrate using a platinum spatula or calcium alginate swab.

### 4.2. Bacterial Isolation and Identification

The samples were transferred into 5 mL of Brain-Heart Infusion broth (Oxoid, Hampshire, UK) and incubated for 24 h at 37 °C. The cloudy broths were inoculated into Columbia CNA blood agar medium (Oxoid, Hampshire, UK) and the agar plates were incubated overnight at 37 °C. After 24 h of incubation, colony growth was observed and identified using a standard method, including Gram staining and catalase production to recognize the *Staphylococcus* strains, and mannitol and coagulase testing to detect the presence of *S. aureus* (Staph Latex Kit; Liofilchem, Waltham, MA, USA). Species-level identification of CoNS bacterial isolates was performed using MicroScan WalkAway 96 Plus automated ID/AST system according to manufacturer’s guidelines.

### 4.3. Antibiotic Susceptibility Test

The *S. aureus* and CoNS isolates were subjected to automatized antimicrobial susceptibility testing performed through MicroScan WalkAway 96 Plus automated ID/AST system as per the manufacturer’s instructions. Antibiotic susceptibility test results were interpreted and bacterial isolates were categorized as susceptible (S), intermediate (I) or resistant (R) according to European Committee on Antimicrobial Susceptibility Testing (EUCAST) breakpoints. The MR and macrolide-lincosamide-streptogramin B (MLSB) Staph*ylococcus* species resistance were detected by the automatized system. The antimicrobial susceptibility for these Gram-positive bacteria was determined using the following antibiotics: ampicillin, amoxicillin-clavulanic acid, cefoxitin, gentamicin, tobramycin, neomycin, chloramphenicol, clindamycin, erythromycin, ciprofloxacin, moxifloxacin, tetracycline and trimethoprim/sulfamethoxazole. American Type Culture Collection (ATCC) 25923 *S. aureus* was used as a control strain.

### 4.4. Statistical Analysis

Statistical analysis was conducted using the IMB SPSS software (version 22.0; IBM SPSS Inc., New York, NY, USA). Descriptive statistics were computerized for the study, and variables such as sex and age were isolated from the study population. Rates of methicillin and MLSB-resistance among *S. aureus* and CoNS strains, cause of ocular infections, were presented as percentages. Antibiotic susceptibility profiles were compared for *S. aureus* and CoNS and were expressed in percentages. A Chi-square test was used to evaluate the relationship between two groups of categorical variables. A *p*-value of ≤ 0.05 was considered statistically significant.

### 4.5. Ethical Consideration Statement.

Ethical approval by the Human Research Ethics Committee was not requested for this study. The resignation was given as our study used laboratory management data and clinical information on patients, collected from databases. This is a retrospective study and not directly associated with patients. This study was consistent with the principles of the Helsinki Declaration.

## 5. Conclusions

The antibiotic resistance trend during the study period was also not encouraging at all. Indeed, mostly among CoNS isolates, a significant increase in methicillin and aminoglycosides rates was recorded between 2017 and 2020, whereas the susceptibility to fluoroquinolones and macrolides remained unchanged but still low. These data suggest that methicillin resistance and multi-drug resistance among ocular *S. aureus* and CoNS isolates should be seriously considered before treating ocular infections in all tested patients. In the future, the isolation, through cultural methods, and the identification of bacterial pathogens followed by the testing of the antimicrobial’s susceptibility profiles will become essential to set up a specific antibacterial treatment and to prevent the development of new resistances. In conclusion, given the high percentage of eye infections caused by *S. aureus* and CoNS and their increasing resistance to various antibacterial agents, the aim of our study is to underline the importance of bacterial susceptibility profiles in order to avoid misuse of antibacterial agents, prevent failure treatments but also at the same time improve the empirical treatment to reduce antibiotic resistance.

## Figures and Tables

**Table 1:** Gender and age distribution of study population.

Gender	n (%)	*p*-Value
Male	155 (48.1%)	-
Female	167 (51.9%)	-
**Age (Years) by Categories**	**n (%)**	***p*** **-Value**
0–30 years old	26 (8.0%)	-
31–60 years old	81 (25.2%)	-
61–90 years old	215 (66.8%)	<0.05 *

* Chi-square test was conducted between the resistance rates for the MR-Cons and MR-*S. aureus* bacterial strains. *p*-value ≤ 0.05 was statistically significant.

**Table 2:** Resistance percentages of different antibiotics between 2017 and 2020 among CoNS and *S. aureus* isolates.

Antimicrobial Agent	% CoNS	% *S. aureus*	*p*-Value
Ampicillin	61.7	23.7	<0.05 *
Amoxicillin-Clavulanic Acid	61.7	23.7	<0.05 *
Cefoxitin	61.7	23.7	<0.05 *
Gentamycin	72.8	41.7	0.05 *
Tobramycin	72.8	41.7	0.05 *
Clindamycin	87.5	56.0	<0.05 *
Erythromycin	81.9	45.8	0.001 *
Tetracycline	58.5	28.6	<0.05 *
Ciprofloxacin	84.2	47.6	<0.05 *
Moxifloxacin	84.2	47.6	<0.05 *
Neomycin	72.8	41.7	0.05 *
Chloramphenicol	0	0	-
Trimethoprim Sulfamethoxazole	0	0	-

* Chi-square was conducted between the resistance rates for the MR-Cons and MR-*S. aureus* bacterial strains. *p*-value ≤ 0.05 was statistically significant.

## Data Availability

Epidemiological data used to support the results of this study are included in the article.
